# Dispersion-corrected extracorporeal arterial input functions in PET studies of mice: a comparison to intracorporeal microprobe measurements

**DOI:** 10.1186/s13550-023-01031-z

**Published:** 2023-09-26

**Authors:** Juela Cufe, Florian Gierse, Klaus P. Schäfers, Sven Hermann, Michael A. Schäfers, Philipp Backhaus, Florian Büther

**Affiliations:** 1 Department of Nuclear Medicine, University Hospital Münster, Albert-Schweitzer-Campus 1, 48149 Münster, Germany; 2 European Institute for Molecular Imaging (EIMI), University of Münster, Münster, Germany

**Keywords:** PET, Input function, Kinetic modelling, Quantification, Dispersion correction

## Abstract

**Background:**

Kinetic modelling of dynamic PET typically requires knowledge of the arterial radiotracer concentration (arterial input function, AIF). Its accurate determination is very difficult in mice. AIF measurements in an extracorporeal shunt can be performed; however, this introduces catheter dispersion. We propose a framework for extracorporeal dispersion correction and validated it by comparison to invasively determined intracorporeal AIFs using implanted microprobes.

**Results:**

The response of an extracorporeal radiation detector to radioactivity boxcar functions, characterised by a convolution-based dispersion model, gave best fits using double-gamma variate and single-gamma variate kernels compared to mono-exponential kernels for the investigated range of flow rates. Parametric deconvolution with the optimal kernels was performed on 9 mice that were injected with a bolus of 39 ± 25 MBq [^18^F]F-PSMA-1007 after application of an extracorporeal circulation for three different flow rates in order to correct for dispersion. Comparison with synchronous implantation of microprobes for invasive aortic AIF recordings showed favourable correspondence, with no significant difference in terms of area-under-curve after 300 s and 5000 s. One-tissue and two-tissue compartment model simulations were performed to investigate differences in kinetic parameters between intra- and extracorporeally measured AIFs. Results of the modelling study revealed kinetic parameters close to the chosen simulated values in all compartment models.

**Conclusion:**

The high correspondence of simultaneously intra- and extracorporeally determined AIFs and resulting model parameters establishes a feasible framework for extracorporeal dispersion correction. This should allow more precise and accurate kinetic modelling in small animal experiments.

**Supplementary Information:**

The online version contains supplementary material available at 10.1186/s13550-023-01031-z.

## Introduction

Kinetic modelling (KM) of dynamic PET allows to quantify molecular features in vivo and to distinguish specific molecular signals from unspecific uptake [1]. This is of particular value in clinical research for investigational tracers, but also for clinical scanning when precise molecular quantification is required. Following the concept of translational research, KM should also be available in small animal PET to establish quantification in preclinical disease models. KM ideally requires not only a precise measurement of the time-activity curve (TAC) from the tissues of interest, but also an accurate dynamic arterial blood tracer concentration. Measurements of this so-called arterial input function (AIF) are already challenging in humans, but are especially problematic in small animal PET. Given the comparatively low spatial resolution of PET, image-derived AIFs have limitations and are biased by partial volume and motion effects. A common alternative in humans is either manual or automated blood sampling via arterial cannulation. In consideration of the lower total blood volume in small animals, arterio-venous shunt systems involving dedicated extracorporeal blood radioactivity detectors have been established to mirror this clinical approach [2–4] allowing KM with preclinical dynamic PET [5]. Recently, we have demonstrated this approach to be applicable in the context of small animal perfusion MRI [6] establishing its use in combined small animal PET-MRI.

Although extracorporeal shunting eliminates the bias of partial volume and motion effects and potentially achieves high signal-to-noise ratios (SNR), delay and dispersion effects are introduced as novel challenges. Dispersion describes a change in the distribution of the arterial tracer blood concentration over time that reflects mixing of the blood, particularly due to interaction with tubing walls, requiring specific correction approaches.

Mathematically, dispersion can be described as a convolution of the true AIF with a suitable dispersion kernel; based on this concept, different models and methods for correcting dispersion effects have been described [7]. However, the adequateness of these correction approaches is a matter of discussion, and validation experiments remain lacking and are difficult to accomplish in small animals.

In this study, we report validation experiments for measurements and dispersion correction of an extracorporeally recorded AIF in mice by simultaneous invasive recordings in the aorta using implanted β-microprobes [8, 9]. To this end, we evaluated the precision of dispersion correction at different extracorporeal flow rates and tested deconvolution with different kernel functions that were derived from radioactivity boxcar function experiments. Finally, we compared the reliability of our dispersion-corrected AIFs in a simulation study of kinetic modelling where the invasively derived AIFs were used as a basis for deriving PET tissue curves.

## Materials and methods

### Experiments

30 mL of human blood mixed with 5000 IU heparin were filled into two beakers where blood was agitated using magnetic mixers and kept at 37 °C. The blood in one beaker was mixed with approximately 100 MBq of [^18^F]F-PSMA-1007. A silicone tube (length: 800 mm, inner diameter: 0.3 mm, outer diameter: 0.7 mm; Reichelt Chemietechnik, Heidelberg, Germany) was connected via T-piece to the two beakers allowing to switch the blood source using vessel clamps. The commercially available Twilite detector (SwissTrace, Menzingen, Switzerland) was used for detecting coincidence annihilation radiation based on two lutetium yttrium orthosilicate (LYSO) crystals in a tungsten casing. Blood passes the sensitive detector volume within a loop and hence twice whilst being pumped through the detector. Scintillation light was separately guided into a photomultiplier unit where signals were processed into coincidence counts per second (coincidence window: 100 ns, temporal resolution: 1 s). The T-piece distance to the centre of the detector was 400 mm. Blood flow was driven downstream by an 8-wheel peristaltic pump (Medorex Schlauchpumpe TL, MDX Biotechnik International, Nörten-Hardenberg, Germany). Chosen flow rates were calibrated by pumping volumes over three minutes and subsequent weighing. The detector response was recorded for three rising-and-falling boxcar functions at six different flow rates F (24, 40, 55, 67, 79, 96 μL/min)

The same set of experiments was conducted with pumping air instead of the non-radioactive blood to determine the dispersion-free geometric sensitivity profile (detector response function) of the Twilite system, accounting for nonzero residence time within in the detector loop.

Female NMRI nu/nu mice (Janvier Labs, Le Genest-Saint-Isle, France), 9–10 weeks old, were housed at a constant temperature (22 °C) and relative humidity (40–55%) under a regular light/dark schedule. Food and water were available ad libitum.

Mice interventions were performed under combined anaesthesia (isoflurane, fentanyl) with temperature maintained at 37 °C ± 1 °C and breathing frequencies kept at 50–65 min^−1^ using typically 1.7–2.3% isoflurane in 100% oxygen.

9 animals were prepared in supine position with two intravenous tail vein catheters (one in each lateral tail vein). 4 µL/g of body mass of a 125 IU/mL heparinised saline solution was injected subcutaneously for anticoagulation. An extracorporeal circulation was applied shunting the surgically accessed femoral artery and a tail vain catheter as previously reported [6]. It consisted of an intravascular polyurethane tube in the femoral artery (length: 10 mm, inner diameter: 0.18 mm, outer diameter: 0.36 mm; Instech, Plymouth Meeting, PA) connected to a 150 mm silicone tube (same type as for boxcar function experiments), a T-piece connector for blood sampling, another 800 mm silicone tube that was placed into the Twilite and further downstream into the peristaltic pump, and additionally featured a glass capillary (length: 10 mm, inner diameter: 0.94 mm) before leading into the tail vein catheter. The distance between the femoral artery and Twilite centre was 400 mm.

**Fig. 1:**
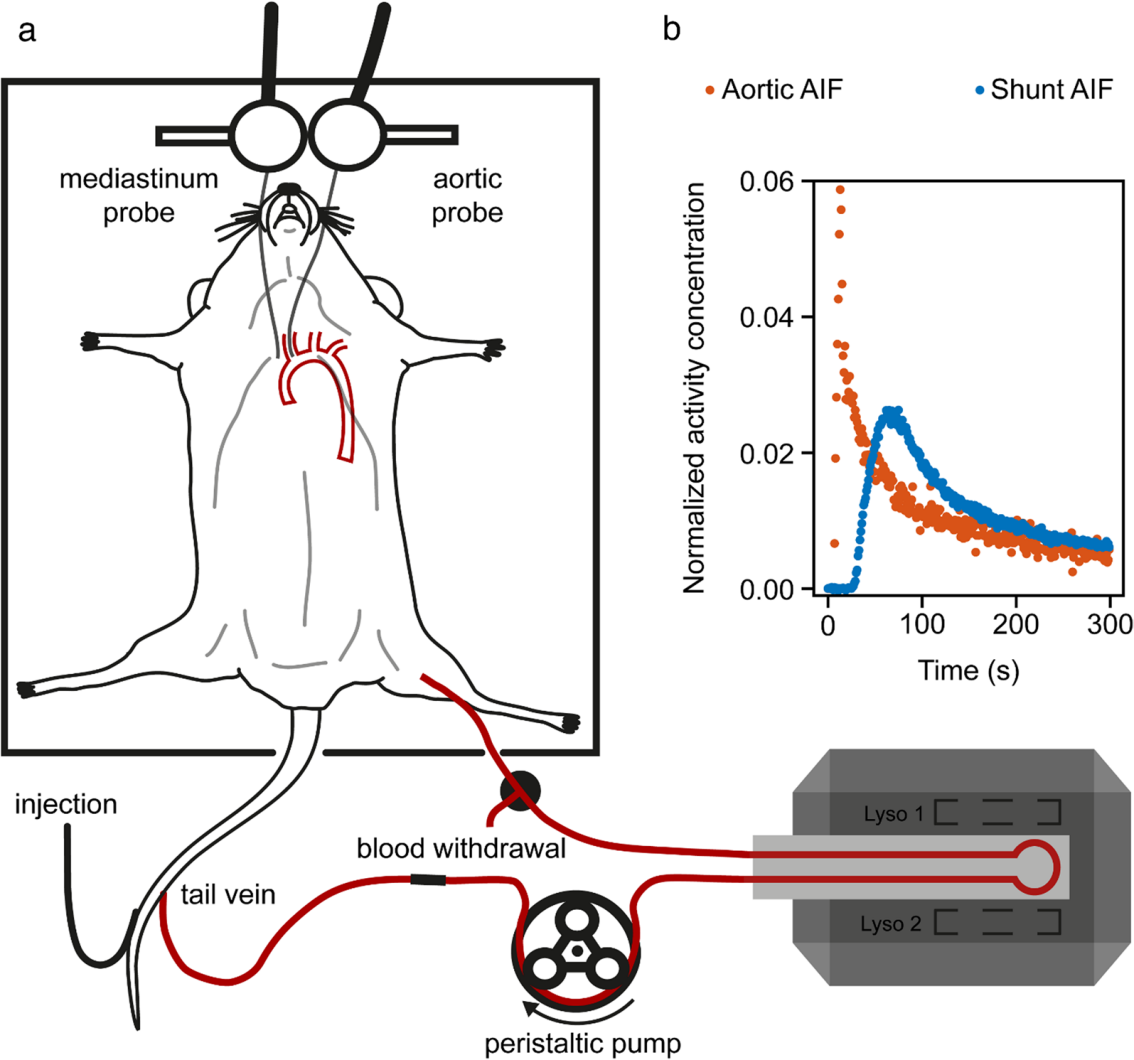
Experimental setup using both a shunt from femoral artery to the tail vein and implanted microprobes for extra- and intracorporeal AIF measurements, respectively **a**; examples of AIF acquisitions demonstrating extracorporeal dispersion **b**

For intracorporeal AIF recordings, a positron-sensitive microprobe (length: 1 mm, diameter: 0.25 mm, coupled to a photomultiplier unit via fiberglass light guides; BioSpace Lab, Nesles-la-Vallée, France) was surgically inserted into the aortic arch via the carotid artery, without occluding the aorta [10]. Another probe was placed in the paraaortic mediastinum via an incision above the sternum. To prevent signal contamination by ambient light, the animal was shielded inside a custom-built opaque plastic chamber. Figure 1 depicts an overview of the experimental setup.

Three mice each were examined for flow rates of approximately 30, 50, 70 µL/min. Constant flow rates were verified using a laser Doppler probe (Perimed AB, Järfälla, Sweden) attached to the glass capillary. 50 μL saline, followed by 100 μL [^18^F]F-PSMA-1007 in saline (39±25 MBq, equivalent to 390 ± 250 MBq/mL) and a 50 μL saline flush were injected using a power injector at 1000 µL/min (Harvard Apparatus, Holliston, MA). Simultaneous microprobe and Twilite measurements were performed for 90 min. Blood samples (approx. 50 μL) were withdrawn at 5, 15, 30 and 90 min post-injection at the T-piece connector for calibration of the microprobes using a calibrated gamma counter (2480 Wizard2, PerkinElmer, Waltham, MA) and a calibrated balance as well as for haematocrit ($hct$) measurements. Afterwards, the silicone tube was cut at the tail vein, and a 3-min sample was taken, to retrospectively calibrate both flow rates and the Twilite detector, the latter again using the gamma counter. Finally, the mice were sacrificed under deep anaesthesia, and the chest was opened to verify correct placement of the microprobes.

### Analyses

All analyses were performed using MATLAB and its nonlinear least-squares solver (MathWorks, Natick, MA).

The acquired Twilite signals were corrected for LYSO background activity and physical decay of ^18^F. The input ${c}_{0}(t)$ into the tube was modelled as a sum of three radioactivity boxcar functions:

1 $${c}_{0}(t)\sim \sum_{i=1}^{3}\left(H\left(t-{t}_{up,i}\right)-H\left(t-{t}_{down,i}\right)\right)$$

with $H$ denoting the Heaviside function, and ${t}_{up,i}$, ${t}_{down,i}$ the switching times between non-radioactive blood/air and radioactive blood, available as tagging information within the detector event list.

The background- and decay-corrected measurement $c(t)$ was modelled as a convolution of ${c}_{0}(t)$ with a suitable kernel $k(t)$:

2 $$c\left(t\right)={c}_{0}\left(t\right)*k\left(t\right)$$

with $*$ denoting the convolution operator. The analysis was conducted considering two different assumptions for the kernel characterisation: a pure dispersion component of the kernel (${k}_{disp}$) describing catheter dispersion alone and a more complex one consisting of the convolution of ${k}_{disp}$ and the detector response kernel ${k}_{det}$: $k\left(t\right)= {k}_{disp}\left(t\right)*{k}_{det}\left(t\right)$. The latter was approximated by a sum of two shifted Gaussians, reflecting that blood passes the sensitive detector volume twice due to the loop:

3 $${k}_{det}\left(t;\mu ,\sigma \right) = \frac{1}{2}\left(\frac{1}{\sigma \sqrt{2}\pi }{\text{e}}^{-\frac{{\left(t-\mu \right)}^{2}}{2{\sigma }^{2}}}+\frac{1}{\sigma \sqrt{2}\pi }{\text{e}}^{-\frac{{\left(t+\mu \right)}^{2}}{2{\sigma }^{2}}}\right)$$

The two parameters $\mu$ and $\sigma$ were determined as functions of the flow rates $F$ by fitting the respective boxcar pulses convolved with ${k}_{\mathrm{det}}(t)$ to the geometric detector response function measurements. Within this parameterisation, $t=0$ refers to the time when radioactive blood reaches the turning point of the loop within the detector unit. As Gaussians are by definition non-vanishing at any time, chosen kernel parameterisation is actually non-causal; however, they are vanishing sufficiently fast so no visually non-causal kernels resulted in any of the investigated cases.

The catheter dispersion kernels ${k}_{\mathrm{disp}}(t)$ were assumed to be described by one of three possible mathematical models:

4a $${k}_{\mathrm{disp}}\left(t\right)={k}_{DG}\left(t;\Delta t,w,{n}_{1},{n}_{2},{\tau }_{1},{\tau }_{2}\right)=H(t-\Delta t)\bullet \left[w\frac{{\left(t-\Delta t\right)}^{{n}_{1}-1}}{{\tau }_{1}^{{n}_{1}}\Gamma \left({n}_{1}\right)} {\text{e}}^{-(t-\Delta t)/{\tau }_{1}}+\left(1-w\right)\frac{{\left(t-\Delta t\right)}^{{n}_{2}-1}}{{\tau }_{2}^{{n}_{2}}\Gamma \left({n}_{2}\right)} {\text{e}}^{-(t-\Delta t)/{\tau }_{2}}\right]$$

4b $${k}_{disp}\left(t\right)={k}_{SG}\left(t;\Delta t,n,\tau \right)=H(t-\Delta t)\bullet \frac{{\left(t-\Delta t\right)}^{n-1}}{{\tau }^{n}\Gamma \left(n\right)} {\text{e}}^{-(t-\Delta t)/\tau }$$

4c $${k}_{disp}\left(t\right)={k}_{ME}\left(t;\Delta t,\tau \right)=H(t-\Delta t)\bullet {\text{e}}^{-(t-\Delta t)/\tau }$$

Here, ME refers to dispersion as convolution with a mono-exponential kernel [11], SG with a single-gamma variate, and DG with a sum of two gamma variates, in order to capture complex dispersion kernel shapes found in some numerical simulations [12]. In particular, three cases were compared:

1. DG: $$k\left(t\right)={k}_{\mathrm{DG}}(t)*{k}_{\mathrm{det}}(t)$$
2. SG: $$k\left(t\right)={k}_{\mathrm{SG}}(t)$$
3. ME: $$k\left(t\right)={k}_{\mathrm{ME}}(t)$$

DG here refers to the most complex model with explicit detector response function modelling, whilst SG and ME assume simpler models without explicit geometric detector response function. Deconvolution was performed by finding optimal kernel parameters for DG, SG, and ME that minimise the sum-of-squared differences between the Twilite measurements $c(t)$ and ${c}_{0}(t)*k(t)$. Model performances were assessed by the Akaike Information Criterion (AIC) [13], by calculating the AIC differences of each method to the smallest determined AIC value [14]. The kernel parameters were parametrised as functions of the blood flow $F$ for the two best models.

For the mice experiments, Twilite and microprobe signals were corrected for background and decay as described above. Twilite data were calibrated to units of Bq/mL using the late 3-min blood sample and normalised to the injected activity concentration, thus resulting in unitless AIF.

The microprobe-based AIF was determined from the background- and decay-corrected aortic signal ${\beta }_{1}(t)$ and mediastinal signal ${\beta }_{2}(t)$ as

5 $${\mathrm{AIF}}_{\beta }(t)= {p}_{0}+{p}_{1}\bullet {\beta }_{1}(t)-{p}_{2}\bullet {\beta }_{2}(t)$$

with constants ${p}_{0}$, ${p}_{1}, {p}_{2}$ being determined from fitting this equation to the 4 blood sample values (in Bq/mL) taken during the experiments. In cases where ${p}_{0}$ was different to zero, the early AIF was set to zero until blood radioactivity was detected by the microprobes. ${AIF}_{\beta }$ was also normalised to the injected activity concentration, resulting again in unitless AIF.

Numerical deconvolution using kernels from the dispersion models was performed by parameterising the Twilite-based AIF as consisting of a linearly rising part followed by a sum of four exponentials [15, 16]:

6 $$\mathrm{AIF}\left(t;{\Delta t}_{0},{\Delta t}_{1},{a}_{i},{\tau }_{i}\right)=\left(H\left(t-{\Delta t}_{0}\right)-H\left({t-\Delta t}_{0}-{\Delta t}_{1}\right)\right)\left(t-{\Delta t}_{0}\right)\frac{{a}_{0}+{a}_{1}+{a}_{2}+{a}_{3}}{{\Delta t}_{1}}+ H\left(t-{\Delta t}_{0}-{\Delta t}_{1}\right)\sum_{i=0}^{3}{a}_{i}{\text{e}}^{-\frac{t-{\Delta t}_{0}-{\Delta t}_{1}}{{t}_{i}}}$$

with ${\Delta t}_{0}$ denoting the onset time, ${\Delta t}_{1}$ the rising time, ${a}_{0}+{a}_{1}+{a}_{2}+{a}_{3}$ the peak height, and ${\tau }_{i}$ the time constants of the exponentials. Nonlinear least squares fitting was performed to provide estimates of the parameters by optimising simultaneously not only the AIF parameters, but also the kernel parameters, to account for potential differences in dispersion between boxcar and mice experiments. Thus, the optimal parameters leading to the best deconvolution are determined as:

7 $${\left({\theta }_{\mathrm{AIF}},{\theta }_{k}\right)}^{\mathrm{opt}}=\underset{{\theta }_{\mathrm{AIF}},{\theta }_{k}}{\mathrm{arg min}}{\Vert \mathrm{AIF}(t;{\theta }_{AIF})*k(t;{\theta }_{k})-c(t)\Vert }^{2}$$

with ${\theta }_{\mathrm{AIF}}=\left({\Delta t}_{0},{\Delta t}_{1},{a}_{i},{\tau }_{i}\right)$ denoting the AIF parameters, and ${\theta }_{k}=\left(\Delta t,w,{n}_{1},{n}_{2},{\tau }_{1},{\tau }_{2}\right)$ and ${\theta }_{k}=\left(\Delta t,n,\tau \right)$ for DG and SG parameters, respectively, resulting in optimal Twilite-based AIF ${AIF}_{DG}$ and ${\mathrm{AIF}}_{SG}$.

Given the high number of optimisation variables and the inner difficulties of deconvolution, prior knowledge and constraints were allowed during the fitting. In particular, since any change in ${\Delta t}_{0}$ can be counterbalanced by a change in kernel $\Delta t$, ${\Delta t}_{0}$ was fixed to 7 s in this process. Moreover, initial values for iterative optimisation related to AIF parametrisation were set to ${\Delta t}_{1}=7 {\text{s}}$,${a}_{0}=0.04$, ${a}_{1}=0.02$, ${a}_{2}=0.01$, ${a}_{3}=0.002$, ${\tau }_{0}=3 {\text{s}}$, ${\tau }_{1}=30 {\text{s}}$, ${\tau }_{2}=300 {\text{s}}$, ${\tau }_{3}=3000 {\text{s}}$, whilst the initial values for the kernel parameterisation were taken from the boxcar function analysis based on the parameterisation of the respective kernel with known blood flow $F$.

AIC analyses were performed as described above in order to decide which deconvolution approach was superior. The peak heights were determined for all three methods and compared using one-way ANOVA. Coefficients of variation (CoV) for the ratios of Twilite-based AIF peak heights to those of ${AIF}_{\beta }$ were calculated. Areas-under-curve (AUC) were evaluated at $t=300 {\text{s}}$ and $t=5000 {\text{s}}$ for all AIF and compared using one-way ANOVA along with multiple comparison test (pairwise test for multiple groups). Finally, average AIFs ± standard deviation (SD) were calculated for the two best kernels.

We simulated PET response TACs using one-tissue compartment (1TC; with compartment concentration ${c}_{1}$) and two-tissue compartment (2TC; with compartment concentrations ${c}_{1}$, ${c}_{2}$) models, assuming that ${\mathrm{AIF}}_{\beta }$ corresponds to the “real” AIF. Additionally, we assumed the radiotracer is not metabolised and not taken up by red blood cells, i.e. blood plasma concentration was equal to ${AIF}_{\beta }(t)/\left(1-\mathrm{hct}\right)$, with the individually determined haematocrit values $hct$. The simulation was conducted using PMOD (PMOD Technologies, Zurich, Switzerland). For 1TC, two parameter sets were chosen: ${K}_{1}=0.200 {\text{min}}^{-1}$, ${k}_{2}=0.100 {\text{min}}^{-1}$ (1TC-sim1) and ${K}_{1}=0.200 {\text{min}}^{-1}$, ${k}_{2}=0.020 {\text{min}}^{-1}$ (1TC-sim2), respectively; for 2TC, two parameter sets were chosen based on literature values for mouse FDG brain PET: ${K}_{1}=0.270 {\text{min}}^{-1}$, ${k}_{2}=0.570 {\text{min}}^{-1}$, ${k}_{3}=0.080 {\text{min}}^{-1}$, ${k}_{4}=0.018 {\text{min}}^{-1}$ (2TC-sim1), and ${K}_{1}=0.140 {\text{min}}^{-1}$, ${k}_{2}=0.190 {\text{min}}^{-1}$, ${k}_{3}=0.070 {\text{min}}^{-1}$, ${k}_{4}=0.005 {\text{min}}^{-1}$ (2TC-sim2), respectively [3, 17]. PET TACs ${c}_{PET}(t)$ were determined as

8 $${c}_{\mathrm{PET}}\left(t\right) = \left(1- {v}_{B}\right){c}_{1}\left(t\right)+{v}_{B}{\mathrm{AIF}}_{\beta }\left(t\right)$$

for 1TC, and

9 $${c}_{\mathrm{PET}}\left(t\right)=\left(1-{v}_{B}\right)\left({c}_{1}\left(t\right)+{c}_{2}\left(t\right)\right)+{v}_{B}{\mathrm{AIF}}_{\beta }\left(t\right)$$

for 2TC, with ${v}_{B}=0.05$, reflecting a typical blood fraction value for soft tissue. These were then modelled using both ${AIF}_{DG}$ and ${AIF}_{SG}$ as AIF, again using PMOD, resulting in fit values for ${v}_{B}$, ${K}_{1}$, ${k}_{2}$, ${k}_{3}$, and ${k}_{4}$, which were compared to the pre-defined simulation values. Total distribution volume ${V}_{T}={K}_{1}/{k}_{2}$ for 1TC and net influx ${K}_{i}={K}_{1}{k}_{3}/({k}_{2}+{k}_{3})$ for 2TC were also compared.

## Results

Optimal ${k}_{det}$ parameter values from the detector response experiments as functions of $F$ were $\mu = 2.00 {\mu L}/F {\text{ and }} \sigma = 0.46 {\mu L}/F + 0.30 {\text{s}}$.

**Fig. 2:**
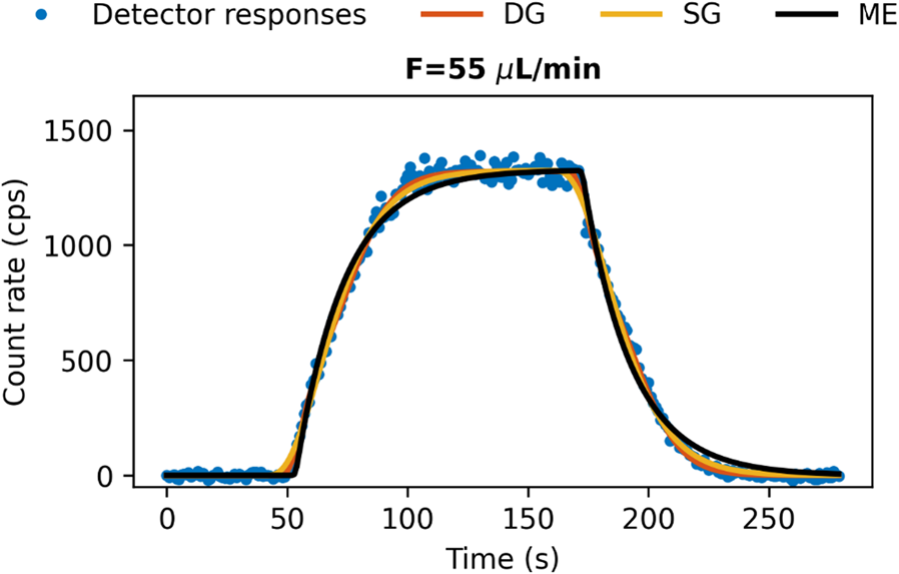
Measured detector response to a boxcar activity profile. Solid lines represent the best fits according to the analysed kernel models at flow rate F = 55 μL/min

**Fig. 3:**
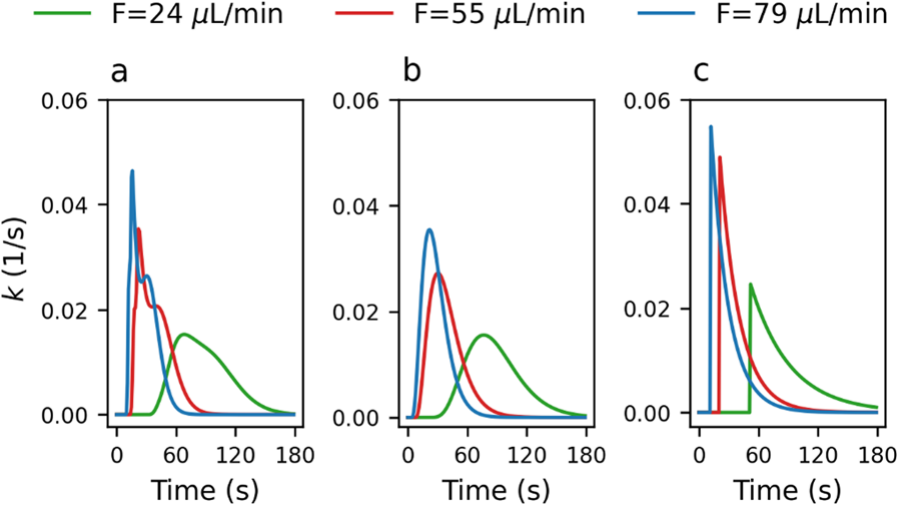
Optimal kernel models for dispersion using DG **a**, SG **b**, and ME **c** for different flow rates

Figure 2 shows an exemplary detector response when switching between non-radioactive and radioactive blood in combination with optimal convolutions of the different kernel models. Optimal kernels for the different models are shown in Fig. 3 for different flow rates $F$. Determined optimal kernel parameters and confidence ranges can be found in the additional file 1: Online Resource S3, S4, S5. Usage of SG demonstrated best fits and lowest AIC values for low flow rates up to $F = 55 {\mu L}/{\text{min}}$. DG was more favourable with better fits and lowest AIC values at higher flow rates. Consistently high AIC values were seen with ME; it was consequently discarded and only DG and SG were subsequently used (Table 1). Both DG and SG kernels were then parameterised as functions of $F$ (Additional file 1: Online Resource S1, S2).

**Table 1:** Dispersion kernel model selection: comparison of ΔAIC and weights

Flow rates $F$ (μL/min)	$\Delta AIC$		
	DG	SG	ME
24	869	0	10,865
40	353	0	6183
55	14	0	19
67	0	80	4774
79	0	326	6559
96	0	12	1929

AIC differences $\Delta AIC$ for the investigated kernel models DG, SG, and ME

In the mice experiments, Twilite background rates were $114.3\pm 0.9 {\text{cps}}$, whilst calibration factors were $3.11\pm 0.48 {\text{kBq}}/{\text{mL}}/{\text{cps}}$ (mean ± SD). Aortic microprobe background was $5.5\pm 1.9 {\text{cps}}$, for the mediastinal probe $4.4\pm 1.1 {\text{cps}}$; calibration values ${p}_{0}$, ${p}_{1}$, ${p}_{2}$ for the unitless normalised AIF amounted to $\left(-2.23\pm 1.26\right)\bullet {10}^{-3}$, $\left(1.41\pm 1.04\right)\bullet {10}^{-4} {\text{cps}}^{-1}$, and $\left(0.64\pm 0.63\right)\bullet {10}^{-4} {\text{cps}}^{-1}$, respectively. These values led to excellent correlations with the blood samples (${r}^{2}=0.994\pm 0.011$).

**Fig. 4:**
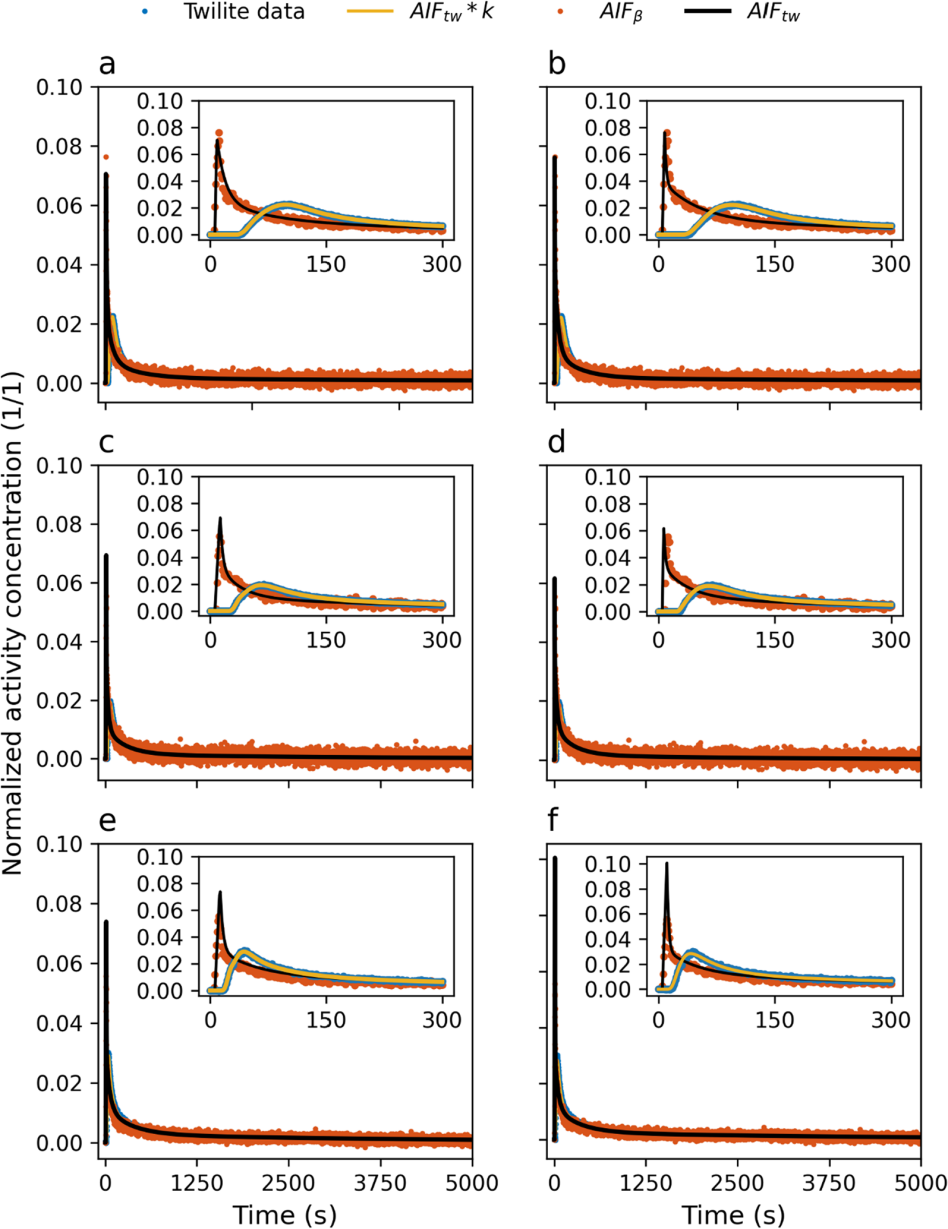
Comparison of deconvolved ${AIF}_{DG}$ (left) and ${AIF}_{SG}$ (right) to ${AIF}_{\beta }$, Twilite measurements, and ${AIF}_{tw}*k$ for three different mice at 32 μL/min **a**, **b**, 53 μL/min **c**, **d**, and 75 μL/min **e**, **f**. Insets show early time behaviour

Deconvolution convergence was found for both kernel models for all measurements. Figure 4 shows a comparison of ${\mathrm{AIF}}_{DG}$ and ${\mathrm{AIF}}_{SG}$ at different flow rates $F$. Visually, both deconvolution with the more complex dispersion kernel DG and the simpler kernel SG gave good fitting results to the Twilite measurements after convolution; all curves demonstrated high similarity between mice experiments. Table 2 shows the results of the AIC analysis; in 9 cases out of 9, ${\mathrm{AIF}}_{DG}$ outperformed ${\mathrm{AIF}}_{SG}$. In addition, residual sum of squares was slightly lower when deconvolution was performed using DG rather than the simple SG.

**Table 2:** AIF model selection: comparison of ΔAIC and RSS

MICE ID	$\Delta AIC$		RSS	
	$AI{F}_{DG}$	$AI{F}_{SG}$	$AI{F}_{DG}$	$AI{F}_{SG}$
#1	0	379.313	0.0195	0.0211
#2	0	30.624	0.1471	0.1482
#3	0	1047.609	0.0100	0.0122
#4	0	1678.458	0.0055	0.0775
#5	0	528.748	0.0316	0.0352
#6	0	211.848	0.0487	0.0464
#7	0	2503.036	0.0091	0.0149
#8	0	611.748	0.0388	0.0439
#9	0	665.410	0.0449	0.0513

AIC analysis for the two deconvolution models ($AI{F}_{DG}$ and $AI{F}_{SG}$; RSS: residual sum of squares for the respective fit)

**Fig. 5:**
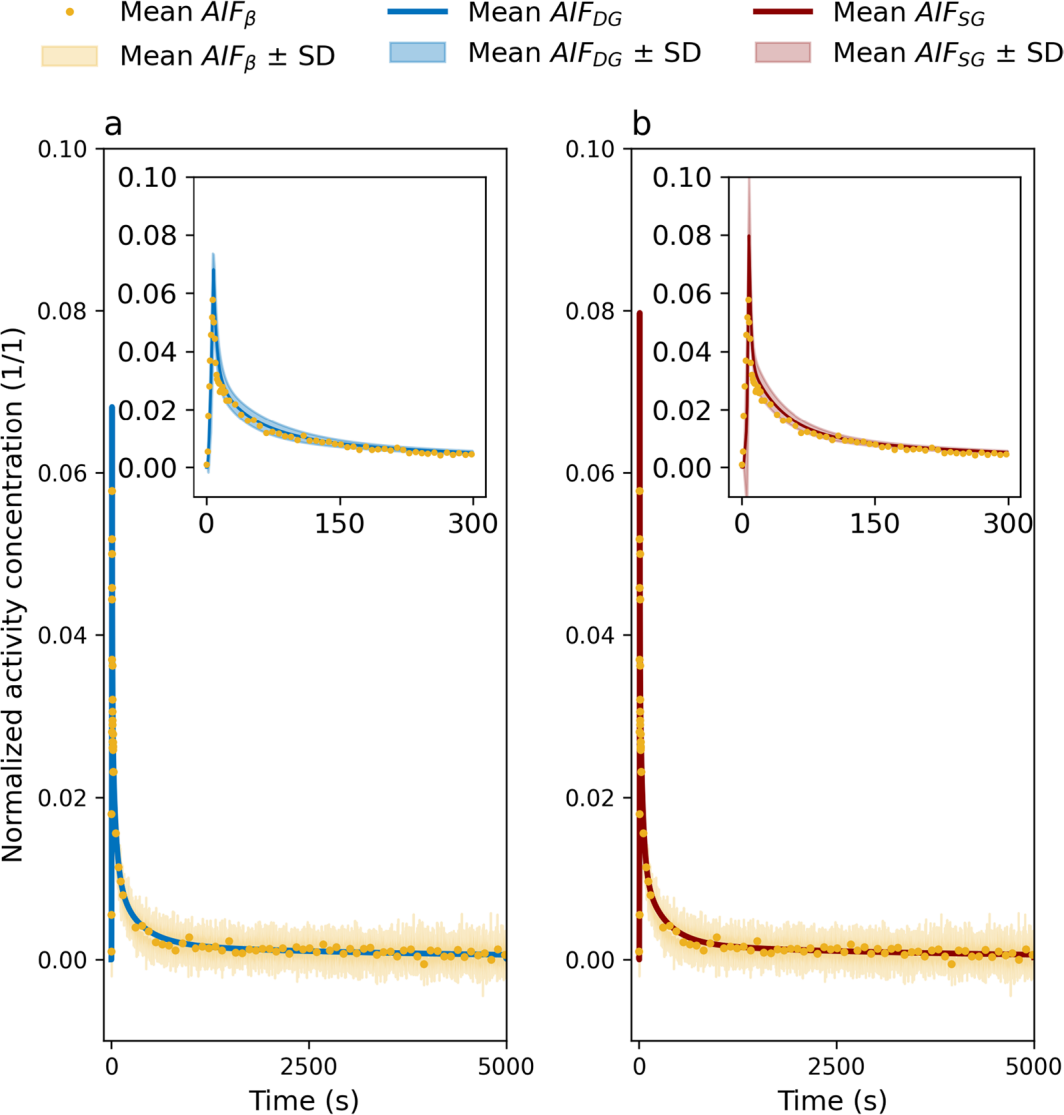
Comparison of population-averaged ${AIF}_{\beta }$ to ${AIF}_{DG}$ **a** and ${AIF}_{SG}$ **b**. Shaded areas refer to average ${AIF}_{\beta }$ ± standard deviation in the main plots. Insets show early time behaviour and shaded areas refer to average ${AIF}_{DG}$ ± standard deviation and ${AIF}_{SG}$ ± standard deviation. Measurement data points have been decreased in the plots for better visualisation of the standard deviation bands

High similarities were found visually between the different averaged AIFs (Fig. 5), which is also apparent in the calculated AUC values (Table 3). Statistical analysis revealed no significant AUC differences between the three different AIF at 300 s ($p=0.18$) and 5000 s ($p=0.83$). The average peak heights for ${\mathrm{AIF}}_{\beta }$ ($0.064\pm 0.011$), ${\mathrm{AIF}}_{\mathrm{DG}}$ ($0.072\pm 0.004$) and ${\mathrm{AIF}}_{\mathrm{SG}}$ ($0.084\pm 0.022$) differed significantly ($p=0.03$) showing that the mean peaks of ${\mathrm{AIF}}_{\beta }$ and ${\mathrm{AIF}}_{\mathrm{SG}}$ were significantly different (*p* = 0.02), whilst CoV of peak height ratios was smaller for $AI{F}_{DG}$ (0.17) than for $AI{F}_{SG}$ (0.29).

**Table 3:** Comparison of areas under the curve for the derived AIFs

MICE ID	${\mathrm{AUC}}_{300 {\text{s}}}$(s)			${AUC}_{5000 {\text{s}}}$(s)		
	${\mathrm{AIF}}_{\beta }$	${\mathrm{AIF}}_{\mathrm{DG}}$	${\mathrm{AIF}}_{\mathrm{SG}}$	${\mathrm{AIF}}_{\beta }$	${\mathrm{AIF}}_{\mathrm{DG}}$	${\mathrm{AIF}}_{\mathrm{SG}}$
#1	2.76	3.47	3.56	10.11	10.78	10.82
#2	3.02	3.86	3.94	7.08	8.84	8.87
#3	3.57	3.67	3.67	10.17	10.33	10.39
#4	2.84	2.89	2.87	7.31	7.02	7.00
#5	3.40	3.66	3.65	9.63	9.37	9.38
#6	3.48	3.18	3.21	10.55	9.94	10.00
#7	2.78	2.96	2.97	7.84	8.89	8.93
#8	3.21	2.96	3.24	7.53	6.92	7.51
#9	3.09	3.78	3.69	10.90	12.50	12.13
Mean ± SD	3.13 ± 0.31	3.38 ± 0.38	3.42 ± 0.36	9.01 ± 1.54	9.40 ± 1.76	9.44 ± 1.60

AUC analysis for $AI{F}_{\beta }$, $AI{F}_{DG}$, and $AI{F}_{SG}$

**Fig. 6:**
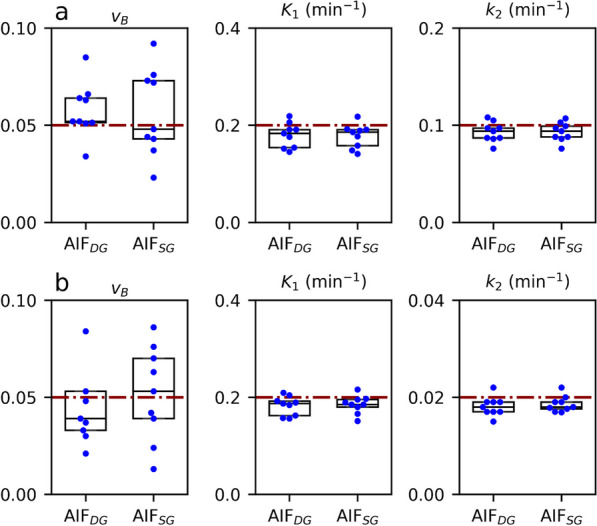
Boxplots of fitted values for the one-tissue compartment models analysed using ${AIF}_{DG}$ and ${AIF}_{SG}$: 1TC-sim1 **a**, 1TC-sim2 **b**; red lines refer to the simulated values

**Fig. 7:**
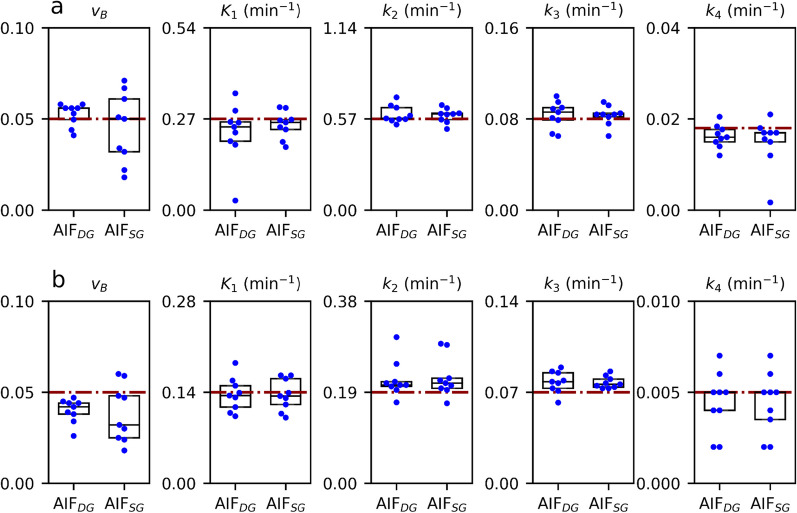
Boxplots of fitted values for the two-tissue compartment models analysed using ${AIF}_{DG}$ and ${AIF}_{SG}$: 2TC-sim1 **a**, 2TC-sim2 **b**; red lines refer to the simulated values

Determined ${v}_{B}, {K}_{1}, {k}_{2}, {k}_{3}, {k}_{4}$ values are shown in Figs. 6 and 7 and summarised in Table 4. In general, the simulated values are well reproduced by both kernel models, with a tendency of slight underestimations of ${K}_{i}.$ The goodness-of-fit ${\chi }^{2}$ was smaller for ${AIF}_{SG}$ in 24 out of 36 modelling cases. Nevertheless, the differences to the pre-defined parameters were small for both approaches.

**Table 4:** Kinetic parameters of the modelling study

Model	Parameter	Simulated value	Fitted value using $AI{F}_{DG}$	Fitted value using $AI{F}_{SG}$	${\chi }_{DG}^{2}$	${\chi }_{SG}^{2}$
1TC-sim1	${v}_{B}$	0.050	0.057 ± 0.014	0.056 ± 0.023	0.899 ± 1.341	0.800 ± 1.302
	${K}_{1}$(min^−1^)	0.200	0.179 ± 0.025	0.177 ± 0.025		
	${k}_{2}$(min^−1^)	0.100	0.093 ± 0.010	0.093 ± 0.009		
	${V}_{T}$	2.000	1.932 ± 0.179	1.910 ± 0.184		
1TC-sim2	${v}_{B}$	0.050	0.050 ± 0.028	0.0517 ± 0.024	0.062 ± 0.092	0.029 ± 0.025
	${K}_{1}$(min^−1^)	0.200	0.182 ± 0.020	0.185 ± 0.018		
	${k}_{2}$(min^−1^)	0.020	0.018 ± 0.002	0.019 ± 0.002		
	${V}_{T}$	10.00	10.135 ± 1.296	9.994 ± 1.136		
2TC-sim1	${v}_{B}$	0.050	0.052 ± 0.006	0.045 ± 0.019	0.211 ± 0.304	0.093 ± 0.163
	${K}_{1}$(min^−1^)	0.270	0.258 ± 0.047	0.253 ± 0.040		
	${k}_{2}$(min^−1^)	0.570	0.600 ± 0.055	0.591 ± 0.045		
	${k}_{3}$(min^−1^)	0.080	0.084 ± 0.012	0.083 ± 0.045		
	${k}_{4}$(min^−1^)	0.018	0.016 ± 0.003	0.017 ± 0.002		
	${K}_{i}$(min^−1^)	0.033	0.031 ± 0.002	0.031 ± 0.003		
2TC-sim2	${v}_{B}$	0.050	0.039 ± 0.007	0.038 ± 0.016	0.014 ± 0.0169	0.023 ± 0.026
	${K}_{1}$(min^−1^)	0.140	0.136 ± 0.026	0.136 ± 0.024		
	${k}_{2}$(min^−1^)	0.190	0.217 ± 0.039	0.221 ± 0.042		
	${k}_{3}$(min^−1^)	0.070	0.078 ± 0.039	0.077 ± 0.042		
	${k}_{4}$(min^−1^)	0.005	0.004 ± 0.002	0.004 ± 0.002		
	${K}_{i}$(min^−1^)	0.038	0.036 ± 0.003	0.035 ± 0.003		

Simulated and modelled parameters along with goodness-of-fits ${\chi }^{2}$ for the investigated compartment models using $AI{F}_{DG}$ and $AI{F}_{SG}$, presented as mean ± SD

## Discussion

Accurate determination of the AIF is at the core of most KM approaches, but difficult in small animal PET, specifically in mice. Extracorporeal measurements in arterio-venous shunts using dedicated detectors have been proposed before [5, 6], but introduce the need for dispersion correction, especially in circumstances where long catheters are required, e.g. when operating in PET-MRI environments [5, 6]. We here evaluated a dispersion correction framework based on different kernel descriptions and compared these with an in mice new method for simultaneous invasive recordings in the aorta using microprobes.

Deconvolution plays a central role in estimating AIFs from extracorporeal measurements, and its ill-conditioned nature can cause problems, especially when managing noisy data, thus high frequency components of the true AIF cannot easily be recovered. A priori knowledge of the shape of the AIF circumvents this problem and therefore a parametric shape model was employed in our study. Furthermore, optimising not only the AIF parameters, but simultaneously also the kernel parameters allowed to include inter-subject variability and errors occurring during the boxcar measurements (e.g. due to imperfect switching between hot and cold blood). Prior knowledge from the experimental data and the boxcar function experiments was used to have good starting points for the fitting routine. Thus, convergence was found for all cases by using both more complex (DG) and simpler (SG) kernels.

In general, both methods achieved good AIF estimation. Visual and quantitative comparison between the different AIF approaches demonstrated satisfying similarities in all individual cases. Quantitative descriptive parameters such as AUC showed similar values for all three different AIFs. Nevertheless, AIF peak heights comparison revealed significant difference between $AI{F}_{\beta }$ and $AI{F}_{SG}$. This particular case seems to be due to the fact that for higher flow cases ($F\sim 70{\mu L}/{\text{min}}$); peak heights were slightly overestimated when using the SG kernel compared to the DG. This is in line with the boxcar experiments, where DG outperformed SG at high flow rates.

Nonetheless, the overall similarity of AIFs was corroborated by the results of simulation and modelling. Here, on average, both $AI{F}_{DG}$ and $AI{F}_{SG}$ could well reproduce the pre-defined values for ${v}_{B}, {K}_{1}$, ${k}_{2}$, ${k}_{3}$, and ${k}_{4}$, respectively.

The dispersion kernels have been found to be well-described by weighted sums of two gamma variates or single-gamma variates [18, 19] rather than previously described less complex models based on mono-exponential kernels [11]. In detail, SG revealed better fits for lower flow rates whilst, on the other hand, the DG model seemed to be more suitable for higher flow rates.

The latter mirrors results presented by Munk et al. [7] proposing a mixed transmission-dispersion model. Here, radioactivity was assumed to be travelling either convectively or interacting with the tubing wall, mathematically treated as an additional compartment, and effectively resulting in a mono-exponential dispersion kernel in addition to the pure convection-based delay. This model is approximately contained in the DG dispersion model in the limit of small dispersion, where one gamma variate can be approximated by an almost delta peak-like distribution (with ${n}_{1}$ approaching 0) and the other reaching a mono-exponential distribution (with ${n}_{2}$ approaching 1). It also agrees qualitatively with simulation studies predicting double peaks in dispersion profiles, which result from the segregation of the solute into the faster-moving region near the centre and the slower-moving region near the wall [12]. This is also similar to findings of dispersion experiments demonstrating flow separations in another AIF recording setup [20]. However, we cannot exclude the possibility that the DG kernel structure is caused by non-dispersion effects occurring during boxcar function experiments.

Compared to the analytical dispersion correction given by Munk et al., the kernel models we use are too complex for a simple inversion, and therefore, we opted for an iterative parametric deconvolution approach, assuming that mouse AIFs have a specific shape in our injection protocol. However, it should be noted that this shape may no longer be suitable for different injection protocols. In these situations, different parametric AIF representations may be used. Promising alternatives, especially when dealing with slower injections, involve modelling the AIF as a convolution of a boxcar function, reflecting the temporal injection length, with a sum of exponentials, as demonstrated in [16].

Using implanted microprobes in vivo have been proposed and described before, with varying degrees of success. Utilising plastic scintillators for positron detection are advisable because of a good sensitivity ratio of positron to gamma events; however, there is still the chance of detecting annihilation photons. Furthermore, placement of a microprobe into a blood vessel of sufficiently large diameter is necessary in order to maximise sensitivity; and yet it still cannot be guaranteed that only positrons released within the blood will be detected. In rat experiments, Pain et al. therefore suggested to use a second microprobe implanted outside the vessel, measuring background radioactivity levels that were then subtracted from the vessel signal [10]. We adopted their approach for mouse scans using four blood samples for calibration to isolate the true aortic blood signal from background radioactivity. As it turned out, non-vanishing values for ${p}_{0}$ led to excellent fits to the blood samples, probably caused by differences in long-range gamma or short-range positron contributions observed by the two probes. However, these influences seem to be comparatively small; we thus believe that the determined $AI{F}_{\beta }$ is a good representation of the true AIF even for times shortly after tracer injection.

There are further limitations to our study. We did not compare this approach with PET image-derived AIF, as our mice experiments were not performed in a PET system due to the spatial constraints when dealing with the microprobe system. Consequently, we could not evaluate the impact on KM with real PET data but used simulations instead. Moreover, we performed only a limited number of experiments at few defined flow rates and only used one tracer, [^18^F]F-PSMA-1007 [21]. However, this tracer should be representative for other small hydrophilic PET tracers regarding its passive pharmacokinetic behaviour and was chosen over [^18^F]FDG because of the lack of specific myocardial uptake, simplifying intracorporeal recordings.

In this study, averaged AIFs served as a means of comparison to the intracorporeal input functions. However, this study cannot be used to evaluate the superiority of individual AIFs over population-averaged AIFs or vice versa due to the limited amount of experimental data.

Validating our approach for integrated PET-MRI KM with non-pump-driven shunts was beyond the scope of this article and will be subject of future work. Furthermore, potential influences of parameters like blood temperature and haematocrit on catheter dispersion was not systematically investigated (but were implicitly taken care of by the additional kernel parameter optimisation in our deconvolution routine).

Despite these limitations, we provide a dispersion-corrected extracorporeal AIF determination framework which we demonstrated to be accurate and ready to use in mouse PET.

## Conclusion

We investigated extracorporeal AIF measurements by performing dispersion correction and successfully used it to calculate dispersion-free AIFs for usage in PET scans of mice. We validated this approach with a novel method for intracorporeal aortic AIF measurements using implanted microprobes. The observed high correspondence of simultaneously measured intra- and extracorporeally determined AIFs and resulting modelling parameters in our simulation study establish our approach as a feasible framework for extracorporeal dispersion correction. This should allow more precise and accurate kinetic modelling in small animal PET experiments.

### Supplementary Information

**Additional file1:** Parameterisation of DG kernels, Parameterisation of SG kernels, Optimal estimates of DG parameters, Optimal estimates of SG parameters, Optimal estimates of ME parameters

## Data Availability

The datasets generated during and/or analysed during the current study are available from the corresponding author on reasonable request.

## Abbreviations

PSMA: Prostate-specific membrane antigen
AIC: Akaike information criterion
AIF: Arterial input function
ANOVA: Analysis of variance
AUC: Area-under-curve
KM: Kinetic modelling
LYSO: Lutetium yttrium orthosilicate
MRI: Magnetic resonance imaging
PET: Positron emission tomography
SNR: Signal-to-noise ratio
TAC: Time-activity curve
