# Supplementary material for: Dispersion-corrected extracorporeal arterial input functions in PET studies of mice: a comparison to intracorporeal microprobe measurements
Source: EJNMMI Res. 2023 Sep 26;13:86. doi: 10.1186/s13550-023-01031-z (PMC10522560; doi:10.1186/s13550-023-01031-z)
Supplement: Supplementary file 1 — Additional file1: Parameterisation of DG kernels, Parameterisation of SG kernels, Optimal estimates of DG parameters, Optimal estimates of SG parameters, Optimal estimates of ME parameters [file 13550_2023_1031_MOESM1_ESM.docx]

# Supplementary Materials

# Dispersion-corrected extracorporeal arterial input functions in PET studies of mice: a comparison to intracorporeal microprobe measurements

Juela Cufe^1,2*^, Florian Gierse^2*^, Klaus P. Schäfers^2^, Sven Hermann^2^, Michael A. Schäfers^1,2^, Philipp Backhaus^1,2*^, Florian Büther^1,2^

^1^Department of Nuclear Medicine, University Hospital Münster, Münster, Germany

^2^European Institute for Molecular Imaging (EIMI), University of Münster, Münster, Germany

^*^Contributed equally to this work


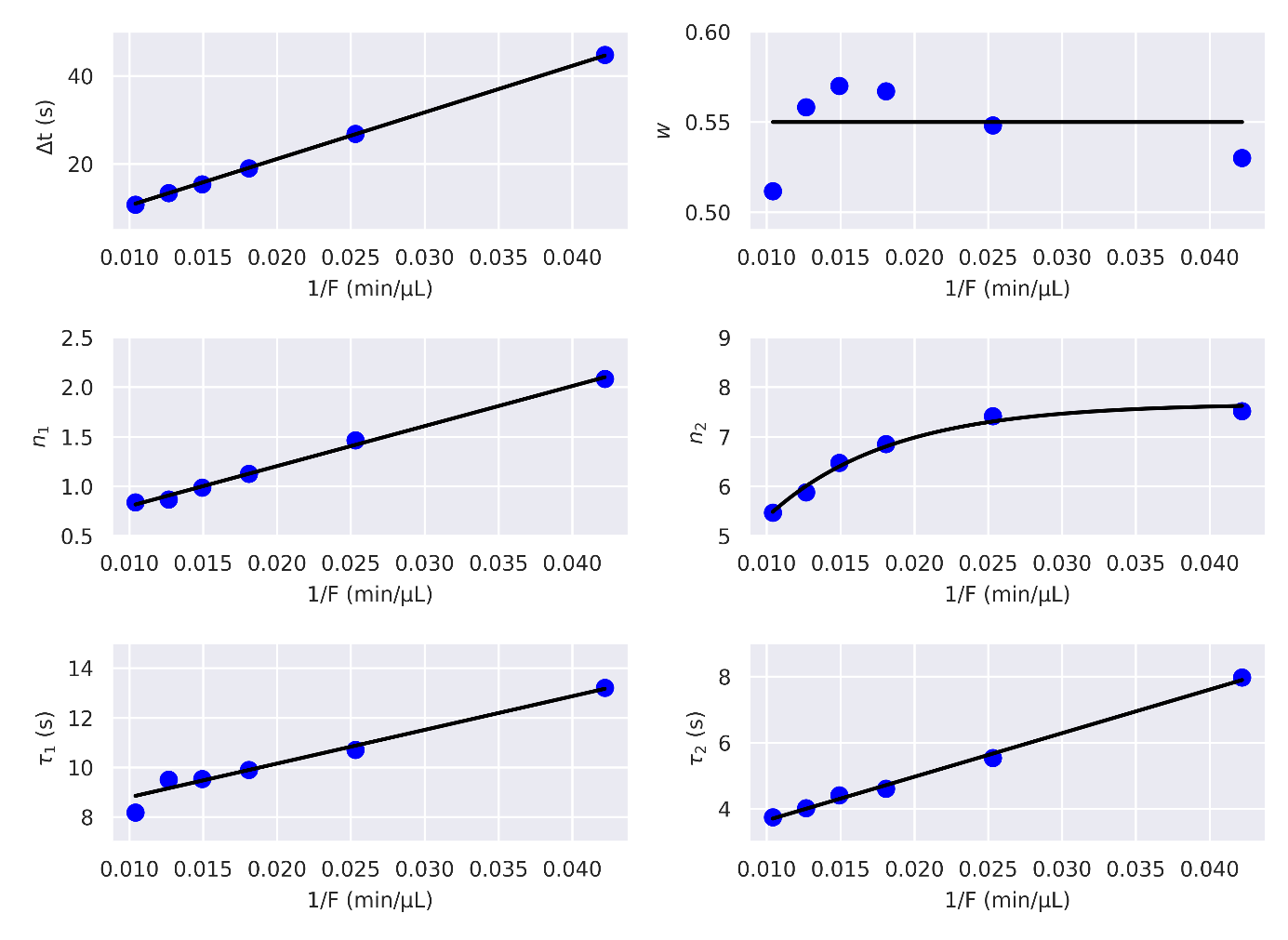


**Online Resource 1:** Determined parameters for kernel model DG (blue dots) as functions of flow rate $F$ and model fits (black lines) parameterised by $F$. Determined optimal parametrisations for the six parameters of DG amounted to: $\Delta t=17.60 \text{μL}/F$, $w=0.55$, $n_{1}=40.36 \text{μL}/{\text{min/}F}+0.39$, $n_{2}=7.67\cdot\left[ 1-\text{exp}\left( -120.59\text{μL}/{\text{min/}F} \right) \right]$, $\tau_{1}=2.26\text{μL}/\text{F}+7.44 \text{s}$, $\tau_{2}=2.20\text{μL}/\text{F}+2.33 \text{s}$.


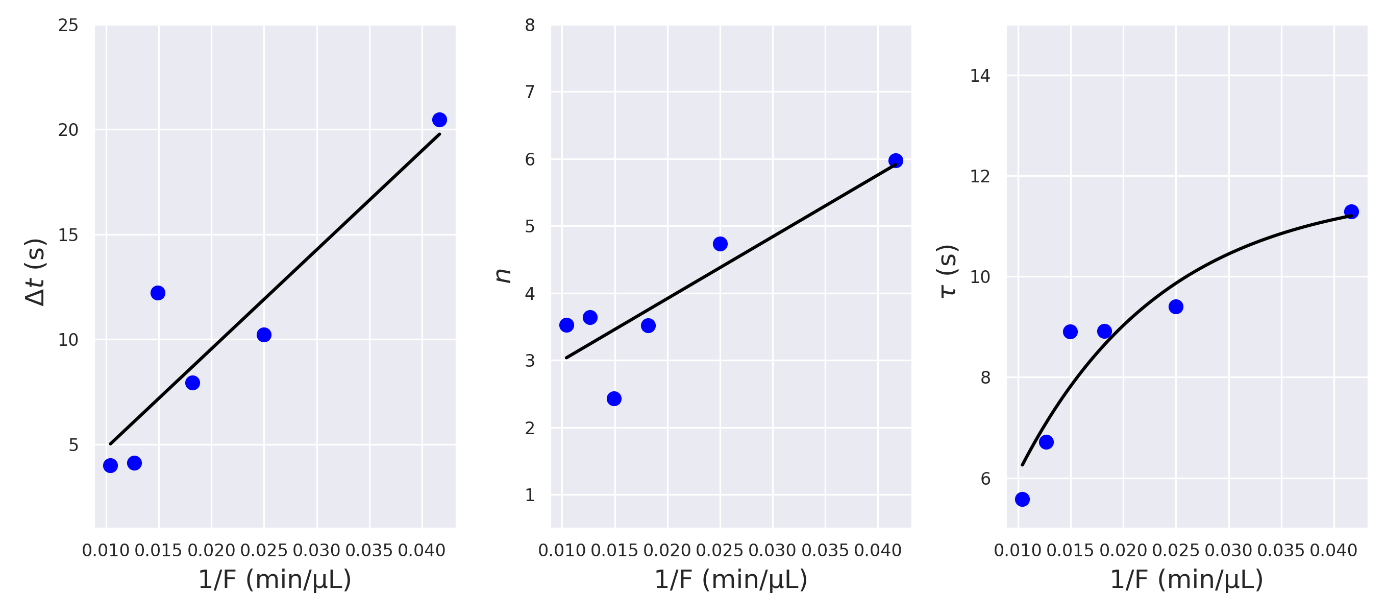


**Online Resource 2:** Determined parameters for kernel model SG (blue dots) as functions of flow rate $F$ and model fits (black lines) parametrised by $F$. Determined optimal parametrisations for the three parameters of SG amounted to: $\Delta t=7.86 \text{μL}/F+0.10 \text{s}$, $n=92.02 \text{μL}/{\text{min/}F}+2.08$, $\tau=11.77 \text{s}\cdot\left[ 1-\text{exp}\left( -72.83\text{μL}/{\text{min/}F} \right) \right]$.

**Table 1** Parameters estimates and confidence intervals for DG

| Flow rates $F$ (μL/min) | WEIGHTED SUM OF TWO GAMMA VARIATES (DG) | | | | |
| --- | --- | --- | --- | --- | --- |
|  | $\Delta t$ (s) | $n_{1}$ | $\tau_{1}$ (s) | $n_{2}$ | $\tau_{2}$ (s) |
| 24 | 44 (24.61, 63.38) | 2.08 (-2.32, 6.48) | 13.09 (-9.8, 35.28) | 7.62 (0.004, 15.22) | 7.82 (3.11, 12.53) |
| 40 | 26.4 (26.38, 26.41) | 1.41 (-0.36, 3.17) | 10.83 (-1.42, 23.08) | 7.30 (2.81, 11.77) | 5.56 (2.76, 8.47) |
| 55 | 19.19 (19.1, 19.3) | 1.07 (-1.82, 3.97) | 9.74 (-10.80, 30.28) | 6.64 (-0.99, 14.26) | 4.72 (0.31, 9.13) |
| 67 | 15.76 (-104.05, 135.57) | 0.99 (-0.33, 2.33) | 9.46 (-69.77, 88.69) | 5.92 (-15.02, 26.87) | 4.29 (0.43, 8.15) |
| 7  9 | 13.37 (-69.86, 96.60) | 0.91 (-18.07, 19.88) | 9.15 (-79.74, 98.04) | 6.00 (-15.46, 27.46) | 3.35 (-10.19, 16.88) |
| 96 | 10.95 (-39.31, 61.21) | 0.94 (-5.85, 7.74) | 8.36 (-11.49, 28.12) | 5.05 (-1.42, 11.54) | 3.69 (1.07, 6.32) |

**Online Resource 3** Optimal parameter estimates and confidence intervals (95%) for kernel model DG for each flow rate $F$.

**Table 2** Parameters estimates and confidence intervals for SG

| Flow rates $F$ (μL/min) | SINGLE GAMMA (SG) | | |
| --- | --- | --- | --- |
|  | $\Delta t$ (s) | $n$ | $\tau$ (s) |
| 24 | 20.47 (1.93, 39.00) | 5.98 (2.55, 9.40) | 11.29 (7.75, 14.83) |
| 40 | 10.23 (-3.34, 23.81) | 4.74 (1.70, 7.77) | 9.36 (6.03, 12.71) |
| 55 | 7.94 (-6.27, 22.14) | 3.52 (0.1, 6.93) | 8.92 (3.89, 13.95) |
| 67 | 12.22 (3.31, 21.14) | 2.44 (0.24, 4.64) | 8.95 (3.97, 13.93) |
| 79 | 4.12 (0.67, 7.56) | 3.64 (2.55, 4.73) | 6.72 (5.56, 7.87) |
| 96 | 3.98 (-3.82, 11.80) | 3.54 (0.54, 6.53) | 5.58 (2.84, 8.33) |

**Online Resource 4** Optimal parameter estimates and confidence intervals (95%) for kernel model SG for each flow rate $F$.

**Table 3** Parameters estimates and confidence intervals for ME

| Flow rates $F$ (μL/min) | MONO-EXPONENTIAL (ME) | |
| --- | --- | --- |
|  | $\Delta t$ (s) | $n$ |
| 24 | 51.00 (-2.36·10^5^, 2.36·10^5^) | 39.55 (38.42, 40.68) |
| 40 | 30.02 (-3.36·10^5^, 3.36·10^5^) | 26.22 (25.21, 27.23) |
| 55 | 20.60 (-1.97·10^5^, 1.97·10^5^) | 20.03 (18.61, 21.45) |
| 67 | 12.70 (-2.27·10^5^, 2.27·10^5^) | 22.46 (20.90, 24.03) |
| 79 | 11.61 (-1.20·10^5^, 1.20·10^5^) | 17.85 (17.50, 18.20) |
| 96 | 12.07 (-1.36·10^5^, 1.36·10^5^) | 12.55 (11.77, 13.32) |

**Online Resource 5** Optimal parameter estimates and confidence intervals (95%) for kernel model ME for each flow rate $F$.
